# Fall Detection Algorithm Using Enhanced HRNet Combined with YOLO

**DOI:** 10.3390/s25134128

**Published:** 2025-07-02

**Authors:** Huan Shi, Xiaopeng Wang, Jia Shi

**Affiliations:** 1 School of Electronic and Information Engineering, Lanzhou Jiaotong University, Lanzhou 730070, China; wangxiaopeng@mail.lzjtu.cn; 2 Department of Mathematics and Physics, Chongqing College of Mobile Communication, Chongqing 401520, China; sjia_nwnu2646@163.com

**Keywords:** fall detection, skeletal key points, YOLOv8, high-resolution network

## Abstract

To address the issues of insufficient feature extraction, single-fall judgment method, and poor real-time performance of traditional fall detection algorithms in occluded scenes, a top-down fall detection algorithm based on improved YOLOv8 combined with BAM-HRNet is proposed. First, the Shufflenetv2 network is used to make the backbone of YOLOv8 light weight, and a mixed attention mechanism network is connected stage-wise at the neck to enable the network to better obtain human body position information. Second, the HRNet network integrated with the channel attention mechanism can effectively extract the position information of key points. Then, by analyzing the position information of skeletal key points, the decline speed of the center of mass, the angular velocity between the trunk and the ground, and the human body height-to-width ratio are jointly used as the discriminant basis for identifying fall behaviors. In addition, when a suspected fall is detected, the system automatically activates a voice inquiry mechanism to improve the accuracy of fall judgment. The results show that the accuracy of the object detection module on the COCO and Pascal VOC datasets is 64.1% and 61.7%, respectively. The accuracy of the key point detection module on the COCO and OCHuman datasets reaches 73.49% and 70.11%, respectively. On the fall detection datasets, the accuracy of the proposed algorithm exceeds 95% and the frame rate reaches 18.1 fps. Compared with traditional algorithms, it demonstrates superior ability to distinguish between normal and fall behaviors.

## 1. Introduction

Falls are the fourth cause of injury in China and the leading cause among the elderly aged 65 and above. Meanwhile, The World Health Organization points out that falls are the second leading cause of unintentional injury worldwide. Timely assistance after a fall can substantially lower the risk of death [1]. With the rapid development of deep learning, computer vision has aroused unprecedented enthusiasm, providing many solutions for fall detection. At present, These algorithms can be divided into two categories: non-computer-vision and computer vision methods.

Fall detection based on non-computer vision uses wearable or environmental sensors to detect fall events. These methods do not rely on digital images and are more suitable for some environments that cannot be monitored by cameras. Wearable sensors usually include accelerometers, gyroscopes, electrocardiograph, etc., which are used to monitor human motion and posture information. Wearable sensors make it easier to achieve personalized monitoring and alarm settings. For instance, Shahzad et al. [2] proposed an automated smartphone fall detection system that employs multi-core learning and the threshold method. Bharathi et al. [3] proposed a fall detection system based on wearable and low-power wireless sensors, which utilizes long short-term memory networks to detect falls among the elderly. Additionally, a multi-layer detection method based on heartbeat packets was proposed by Ning et al. [4], which improves network stability and reduces packet loss rates by collecting information on daily activities and fall states using acceleration, direction angle, and gyroscope sensors. The information is uploaded via smartphone, and the accuracy of algorithms exceeds 85% in detection. The fall detection algorithm based on smartphones has high popularity, and users do not need additional devices. However, since the smartphone’s carrying position is non-fixed, it can easily affect accuracy. Moreover, the detection target is limited to the wearer. Wearable devices are also prone to being forgotten or lost.

In contrast, for the fall detection algorithm based on smartwatches, the wearing position is relatively fixed, resulting in more stable detection data. It is also convenient to wear and can perform real-time monitoring. Ngu et al. [5] designed a fall detection system based on smartwatches combined with a transfer learning strategy, which solved the problems of false alarms caused by insufficient data during fall detection and model drift across different devices. Ngu et al. [6] designed a fall detection system that uses a collaborative edge–cloud framework to run personalized deep learning in real-time on smartwatch devices. In addition, the team designed a suitable UI to implement fall detection on the watch screen. The study elaborately presented the collaborative software architecture and demonstrated how the system automates the fall detection pipeline. Mauldin et al. [7] designed the SmartFall application for the Android platform, which used an IoT-based smartwatch to collect accelerometer data for fall detection. Additionally, the study introduced a three-layer open IoT system architecture for SmartFall, which could be applied to the collection and analysis of other sensor modalities. Şengül et al. [8] developed a smartwatch-based fall detection system. The system transmits sensor data to the cloud via a mobile application, employs the Bica interpolation method to increase the number of samples, and uses a bidirectional long short-term memory (BiLSTM) network to classify and recognize the 38 collected features. The designed system demonstrates excellent detection performance. Mohan et al. [9] proposed a fall detection system based on AI and IoT for the elderly. It uses wearable sensors to collect real-time data and machine learning models for analysis, enabling efficient and accurate detection. But this system has limited battery life and requires frequent charging.

Environmental sensors are utilized to monitor changes in the surrounding environment, including pressure, infrared, and vibration sensors. By analyzing data from these sensors, it is possible to identify fall or loss of balance events. Environmental sensors can effectively capture the location of fall events in specific areas. Wang et al. [10] proposed a fall detection method utilizing convolutional neural networks (CNNs) [11] with adaptive channel selection for ultra-wide-band (UWB) radar signals. This approach acquires data from UWB radar, separates noise from useful signals using adaptive channel selection, and trains a fall recognition model by fusing features from frequency-domain and time-domain images. Zhou et al. [12] introduced a fall detection method based on deep learning and multi-sensor fusion. It uses radar and cameras to capture human posture with short-time Fourier transform. Finally, it uses multiple CNNs to judge whether a fall has occurred. However, environmental sensor-based fall detection methods can only monitor falls in specific areas, are costly, and are inconvenient to relocate. Additionally, false alarms easily occur due to non-fall events.

Fall detection based on computer vision detects fall events by capturing and analyzing digital images of humans. This method usually involves the use of deep learning algorithms, such as CNNs, recurrent neural networks (RNNs) and long short-term memory (LSTM) [13] networks, to extract features and recognize patterns from images, thereby identifying potential fall-related characteristics. By monitoring real-time video streams and integrating pre-trained models, it can automatically detect accidental falls and trigger alarms, enabling timely rescue measures. Liu et al. [14] proposed a fall detection algorithm based on three key points, which involves extracting the feature centers of the head, torso, and feet. The algorithm calculates the distance ratios between the head and torso and between the torso and feet, as well as the angle between the tilt vector and the horizontal plane, to detect falls. However, extracting human contours from grayscale images is highly susceptible to clothing interference. Nogas et al. [15] redefined the fall detection problem as an anomaly data detection problem, where the algorithm detects falls from data captured by thermal imaging or depth cameras by utilizing deep spatiotemporal convolutional autoencoders and reconstruction scores across time window frames. This approach transforms the fall detection task into a binary classification problem cleverly. Shu et al. [16] proposed a vision-based intelligent system for detecting human behavior, using the YOLOv5s detector to recognize three behavioral states: falling, sitting, and walking. However, lying down is an inevitable daily behavior, which increases the misjudgment rate when lying-down behavior is included in the detection scope. Rougier et al. [17] adopted foreground segmentation technology to extract historical images of human motion from video sequences and determine whether a fall has occurred by analyzing changes in body posture. In addition, Yuan [18] and Wang et al. [19] proposed a multimodal visual monitoring fall detection algorithm, which is implemented by integrating adaptive filtering with an improved feature extraction network in factory workshop scenarios. Han et al. [20] proposed a key-point data augmentation method for human pose estimation, which improves the model’s ability to perceive occluded key points and enhances the performance of pose estimation algorithms in occluded scenarios. Obviously, fall detection methods based on deep learning require extensive training, leading to high time costs. To ensure the algorithm’s accuracy and generalization ability, a large amount of fall event data covering diverse scenarios, populations, and postures needs to be collected. This not only consumes significant human and material resources but also raises privacy concerns.

As mentioned above, although great progress has been made in fall detection algorithms, the following challenges still exist:

- In complex scenarios, object detection algorithms typically encounter problems with insufficient feature extraction. When it comes to fall detection, a more precise extraction of human contours is required. As a result, the issue of insufficient feature extraction has become a bottleneck in the development of fall detection technology.
- The YOLO series networks encode the input image into a low-resolution representation via a sub-network, then obtain target features through a sequence of high-to-low resolution convolutions (e.g., ResNet [21], VGGNet [22]), and finally recover high-resolution representations from the encoded low-resolution features. Notably, the high-to-low resolution image processing inevitably causes spatial information loss.
- Human pose estimation technologies primarily focus on precise key-point localization in unoccluded scenarios, thereby overlooking the occlusion problem during human image acquisition.

To address the aforementioned issues, this paper makes the following contributions:

- First, we apply random rectangular occlusions to images for data augmentation, training the model with both occluded and unobstructed data to improve its generalization capabilities.
- Second, by performing lightweighting operations and applying an attention mechanism, we improve the YOLOv8 network to increase the accuracy of target extraction and obtain the location information for each human figure in the image.
- Third, we incorporate an attention mechanism into the key-point detector, which increases the accuracy of human key-point detection and obtains key-point positions.
- Finally, we analyze the information on the position change of skeletal key points, the decline speed of the center of mass, the angular velocity between the trunk and the ground, and the height-to-width ratio of the body, using these factors as the basis for judging falls. Moreover, we design a voice mutual confirmation mechanism to improve the accuracy of fall judgment.

The rest of this work is organized as follows. The Section 2 describes a detailed review of existing fall detection algorithms. The Section 3 describes the proposed algorithm’s structure and implementation process. The Section 4 outlines the experimental setup and data sources, including ablation experiments to test the proposed algorithm and a comparative analysis of the proposed method against other existing methods. And the Section 5 concludes with a summary of the research, demonstrating the practical significance of the proposed algorithm for fall detection tasks in the real world.

## 2. Related Work

The object detection algorithms based on CNNs are the mainstream research in the field of object detection. Researchers are committed to enhancing the generalization ability of the model to unknown data. A crucial step is data augmentation in the process, which involves transforming and processing the original data to generate new training samples, thereby expanding the datasets and improving generalization performance. For instance, data augmentation includes random rotation, flipping, scaling, cropping, background perturbation, and data synthesis.

Human pose estimation consists of key-point detection, key-point connection, and pose inference. It aims to detect and identify specific body parts, such as the head, shoulders, elbows, and knees, and to accurately infer and analyze the body’s pose by locating the positions of these key points. Pose estimation algorithms are generally categorized into bottom-up and top-down approaches.

Bottom-up pose estimation algorithms directly detect all human key points in an image and then group and assign these key points to corresponding individuals using strategies based on distance, angle, or deep learning approaches. Cao et al. [23,24] utilized partial affinity fields to learn the relationships among key points. By encoding global contextual information and allowing bottom-up parsing operations, this method achieves real-time multi-person key-point detection. Bian et al. [25] proposed a fall detection method using a rapid decision forest to extract the skeleton. And before making decisions, they correct the direction of the human frame by frame and track head position changes to improve fall detection accuracy. Insafutdinov et al. [26] presented an indoor fall detection method that combines multi-camera collaboration with a key-point detector. The method performs collaborative computations based on shared image information to identify falls. Angelini et al. [27] introduced a behavior recognition method based on the OpenPose model. They classified human skeletal key-point features into low-level and high-level features, which are respectively fed into an LSTM and a one-dimensional vector for pose recognition. In bottom-up pose estimation algorithms, key-point detection does not rely on initial object detection, thereby reducing processing time and performing well in multi-person scenes. However, the detection accuracy of this algorithm declines in dense or overlapping human scenarios.

In contrast, the top-down pose estimation algorithm identifies the overall human body region through the object detector or human body detector and then finds and identifies the key points belonging to each person in these regions one by one. Raza et al. [28] deployed the YOLOv4-tiny object detector on the OAK-D edge device, using OpenVINO for weight conversion to achieve multi-person fall detection in sample scenes. Bo et al. [29] compared the performance of the YOLOv5n, YOLOv5s, and YOLOv6s network models for three human behaviors, namely fall, sit, and walk. The comparison concluded that YOLOv5s has the best detection performance with 94.8% fall detection accuracy. Wang et al. [30] proposed a fall detection method using dual-channel feature fusion, dividing fall events into falling and fallen. The method utilized the YOLO model to distinguish static fall behaviors and employed the OpenPose model to obtain the positional information of human key points to analyze dynamic features. Multi-layer perceptron (MLP) and random forest were used to classify the dynamic and static feature data, respectively, and then the classification results were combined to achieve fall detection. Wang et al. [31] raised the issue of feature map extraction in CNNs, where information is lost during the transformation from high resolution to low resolution and then back to high resolution. They proposed a method to maintain high-resolution feature representations throughout the entire process. This is achieved through parallel transmission of convolutional streams at the same resolution and serial connections of convolutional streams from high to low resolution, enabling information exchange between different resolutions and improving the accuracy of pose estimation. However, this high-resolution network is only suitable for single-person detection and requires an object detector for multi-person scenarios. More importantly, the top-down pose estimation algorithm aligns with the intuitive human visual processing pattern: from whole to local. The algorithm first determines the overall human body position and then detects detailed key points, enabling more accurate key-point localization and association. Nevertheless, this approach heavily relies on the object detector, and if human detection is inaccurate, it will compromise the accuracy of subsequent fall judgments.

Falls are more likely to occur in multi-person scenarios with occlusions in daily life, such as sports fields, rehabilitation centers, and nursing homes. In these environments, people may obscure each other. Although bottom-up detection methods are faster, they may cause errors in individual skeletal connections in dense crowds or even misalignment of key points between different people. However, top-down pose estimation algorithms align with the intuitive pattern of human visual cognition—from whole to local. The algorithm first determines the overall human body position, then performs detailed key-point detection, thereby enabling more precise key-point localization, reducing errors in key-point connections between individuals, and improving the detection accuracy of human dynamic key points to a certain extent. Therefore, we choose to study the top-down method for fall event detection.

**Figure 1:**
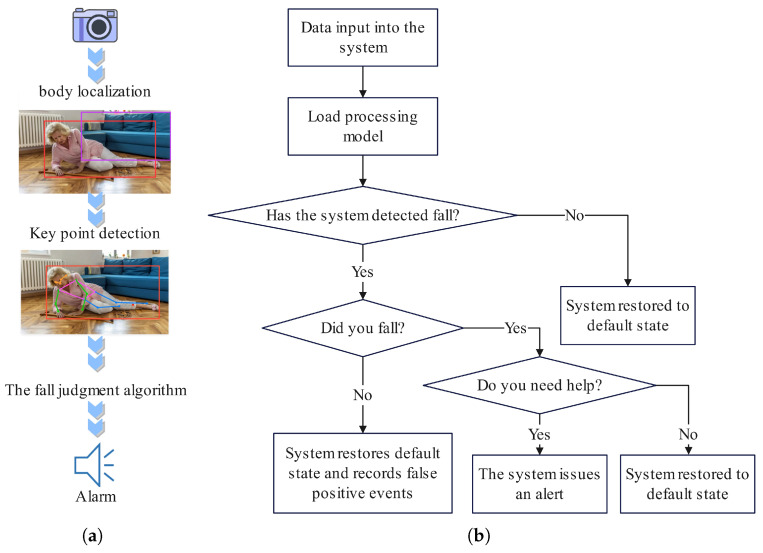
The architecture of the fall detection system is as follows: After capturing images via a camera, the system employs the improved YOLOv8 for human body localization to obtain bounding box information and then uses the BAM-HRNet network to extract key-point details. By analyzing the dynamic trends of key points, the system determines whether a fall has occurred. Upon detecting a fall event, it triggers the voice inquiry module to verify whether assistance is required. (**a**) System overview. (**b**) The processing flow of the system after detecting a fall.

In complex scenarios, YOLOv8 can effectively leverage global contextual information to precisely perform real-time detection and localization of individuals in images, laying a foundation for the subsequent key-point detection module to accurately identify key points. Given the strict requirements for detection accuracy in fall detection tasks, acquiring precise positional information of human key points is of vital importance. Traditional key-point detection methods typically encode input image feature maps into low-resolution representations [32] and then attempt to restore them to high resolution. Inevitably, this process leads to the loss of partial spatial information during the encoding of images into low-resolution features, resulting in inaccurate feature extraction. In contrast, HRNet [32] maintains high-resolution features throughout the entire network training process, a characteristic that enables it to capture fine-grained details more efficiently. Additionally, HRNet employs a multi-scale feature fusion strategy, which not only improves the accuracy of pose estimation but also enhances its robustness. In summary, this paper proposes a top-down fall detection algorithm that combines the improved YOLOv8 and BAM-HRNet for key-point extraction. By leveraging the advantages of these two technologies, the accuracy and robustness of fall detection are significantly enhanced. The architecture of the fall detection algorithm is shown in Figure 1.

## 3. Materials and Methods

### 3.1. Experimental Equipment and Datasets

#### 3.1.1. Experimental Equipment

The software environment of experiments is PyCharm 3.9 and the hardware environment is NVIDIA GeForce RTX 4060 Laptop GPU, 12th Gen Intel® Core™ i7-12650H.

#### 3.1.2. Datasets

There are mainly three types of datasets used in this study: object detection datasets, key-point detection datasets, and fall detection datasets.

- Object Detection Datasets

The object detection network was trained on the COCO train2017 object detection datasets [33] and Pascal VOC 2007 datasets [34] to ensure the model can accurately identify and localize human bodies.

B. Key-Point Detection Datasets

The key-point detection module utilized the COCO train2017 key-point datasets, with 64,115 images for training and 2693 images for validation, ensuring the network can precisely extract dynamic key-point information. Additionally, it was tested on the OCHuman human key-point datasets [35] containing 5000 images with partial occlusions.

C. Fall Detection Datasets

The datasets for fall detection consist of two public datasets and a self-built dataset. The Fall Detection Dataset (FDD) [36] includes approximately 20,000 images of different postural behaviors simulated by 5 participants indoors, covering various typical action scenarios. The UR Fall Detection Dataset (URFD) [37] contains 30 fall events and 40 daily life behavior image sequences, focusing on indoor scenarios for the elderly living alone. The self-built datasets target outdoor public spaces, comprising 1307 fall images in diverse scenarios and 50 videos with different fall postures, complementing the public datasets by addressing scene diversity and establishing a more comprehensive cross-scenario testing framework. The datasets are labeled using the Make Sence online labeling platform.

### 3.2. The Proposed Algorithm

#### 3.2.1. Data Pre-Processing

**Figure 2:**
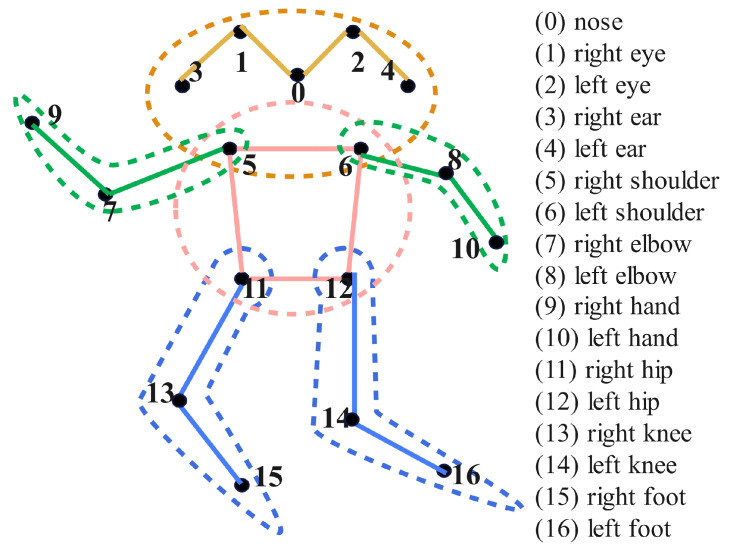
The illustration of key points and division of grouped training. The different color indicates the different group. Blue, pink, green and brown respectively denote the training groups of key points for legs, upper body, arms and head.

To better enable the model to learn the connection of human key points, the 17 human key points are grouped into four categories for training: head, arms, body, and legs. The numbering of key points and their corresponding body parts are illustrated in Figure 2. The experimental results are shown in Table 1, the results indicate that group training can enhance the model’s ability to learn the connection relationships of key points. By constraining the human body, the proposed algorithm enhances the perception of relationships between key points, which facilitates the model to locate human key points in occluded scenarios successfully.

To better deal with the issue of detection failures due to occlusions, random erasing [38] is applied before training to improve the generalization ability of network to various levels of occlusion. Random erasing involves occluding an image with rectangular boxes of arbitrary size at random positions, replacing the occluded regions with the average pixel value of ImageNet [39], and generating new image data. Random erasing can be categorized into three types:

(1) Random occlusion on the entire image (Image Random Erasing, IRE).

(2) Random occlusion of key points within the bounding box of the detected human, provided that the bounding-box prediction is successful (Key-Point Random Erasing, KRE).

(3) Simultaneous random occlusion on the entire image and on the key points locations (Image/Key-Point Random Erasing, I-KRE).

During training, the degree of occlusion is determined for the proportion of the area covered by the rectangular box. Therefore, choosing an appropriate occlusion ratio is an important step.

**Figure 3:**
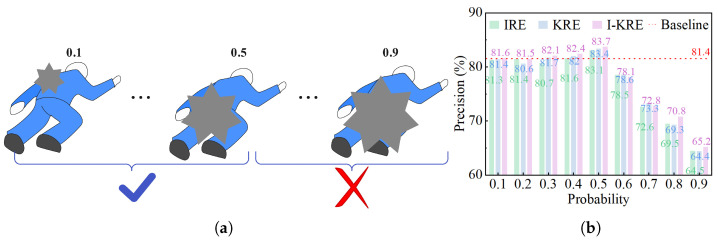
The illustration of different random erasing levels. (**a**) Example of occlusion ratio. When the occlusion factor is in the range of 0.1–0.5, the performance will increase. (**b**) Comparison results of different random erasing levels. On the COCO datasets, the comparison results of key-point detection accuracy obtained by performing IRE, KRE, and I-KRE operations with rectangular blocks of different sizes.

Figure 3 shows examples of random occlusion ratios and the experimental results for three occlusion methods at different occlusion ratios. The results show that the optimal performance is achieved when the erasing ratio is 0.5.

#### 3.2.2. Network Structure Design

- Object Detection Approach

**Figure 4:**
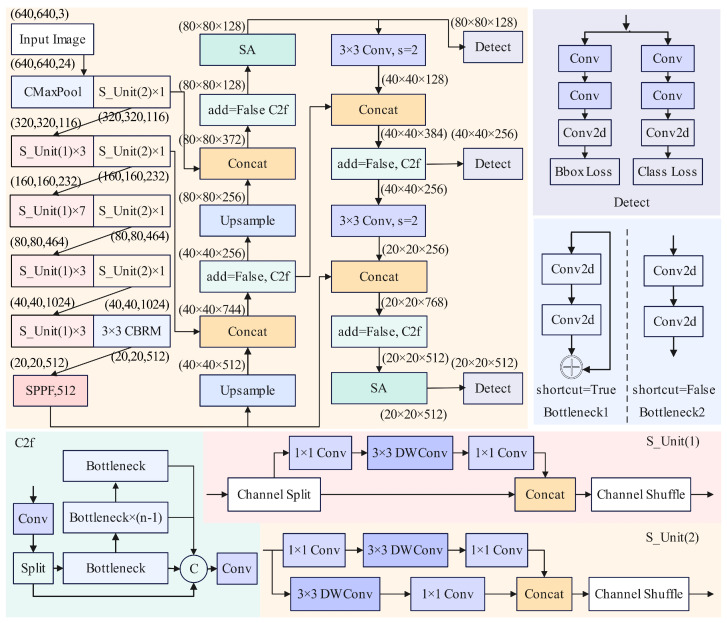
Improved YOLOv8 network architecture. The backbone of improved YOLOv8 utilizes ShuffleNetV2 to achieve lightweight feature extraction. S_Unit(1) is the basic unit. S_Unit(2) is the spatial down-sampling unit. The mix attention mechanism (SA) is added to the neck. In the process of extracting detailed features, it connects across levels to enhance the relevance of context information.

Figure 4 shows the network structure of the improved YOLOv8. The ShuffleNetV2 [40] network, which has channel shuffle, point-wise group convolution, and consists of basic units with depth-wise separable convolutions and down-sampling units, serves as the lightweight backbone of the improved YOLOv8.

The backbone has channels of 24, 116, 232, and 464. This design effectively reduces computation and parameters while enabling initial feature extraction. Additionally, the C2f module, which inherits the ELAN cross-stage connection idea from YOLOv7 [41], enriches gradient flow information. The basic unit named S_Unit(1) splits channels into two branches in any order. One branch remains unchanged, while the other first performs a 1 × 1 convolution to change the number of channels, followed by a 3 × 3 depth-wise separable convolution to better extract features. Subsequently, another 1 × 1 convolution adjusts channel dimensions to ensure both branches have the same number of channels. Finally, the two branches are fully connected, and a channel mixing operation is performed to enable information sharing across channels. The down-sampling unit named S_Unit(2), with a stride of 2 in the depth-wise separable convolution, increases the receptive field, halves the feature map size, doubles the number of channels, and applies channel shuffle to ensure thorough communication and utilization of different channels.

Only when the algorithm accurately captures subtle changes in the human body can the system better distinguish between elderly individuals’ daily activities and accidental falls. The improved backbone network produces feature maps of size 20 × 20, which are fed into a serial Spatial Pyramid Pooling Fast (SPPF) module [42]. This module implements max pooling with scales of 5, 9, and 13. During the construction of the entire network model, cross-stage connections are employed to prevent gradient vanishing or explosion, enhance the network’s feature extraction capability, and improve the accuracy of localization information.

**Figure 5:**
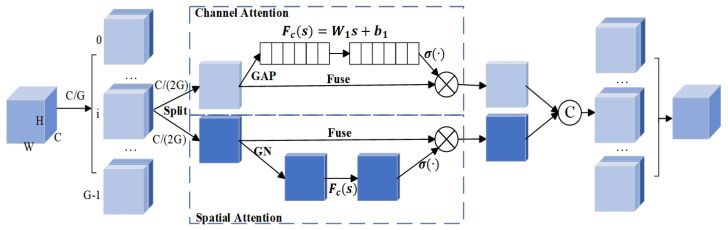
The network architecture of SA. The SA mechanism, also known as group attention mechanism, performs spatial feature extraction and channel information enhancement processing on sub-features separately and finally mixes and fuses the features of each branch. The number of groups is 2 in the study, i.e., G=2.

At the same time, adding the mix attention mechanism is named Shuffle Attention (SA) [43,44] to obtain the information of channels. The structure of SA is shown in Figure 5. In the improved network, the SA mechanism processes feature maps as follows: Let the feature map output by the network backbone be $I\in R^{C\times H\times W}$, where *C* represents the number of channels, and *H* and *W* are the height and width of the feature map, respectively. The feature map *I* is divided into two groups for parallel processing. After grouping, denote the two sub-feature groups as $I_{1}$ and $I_{2}$. The number of channels is $\frac{C}{2}$ in each sub-feature group, i.e., $I_{1},I_{2}\in R^{\frac{C}{2}\times H\times W}$.

Each of the sub-feature groups $I_{1}$ and $I_{2}$ is further divided into subgroups. After the second-stage grouping, the number of channels becomes $\frac{C}{4}$ in each sub-branch. Suppose the sub-branches obtained from the second-stage grouping of $I_{1}$ are $I_{11}$ and $I_{12}$, and those from $I_{2}$ are $I_{21}$ and $I_{22}$. Then, $I_{11},I_{12},I_{21},I_{22}\in R^{\frac{C}{4}\times H\times W}$. One of the branches (e.g., $I_{11}$ and $I_{21}$) is used to capture channel information. Through a specific channel attention operation, important information in the channel dimension of the feature map is mined. The other branch (e.g., $I_{12}$ and $I_{22}$) is used to acquire spatial information. A spatial attention mechanism focuses on important regions in the spatial dimension of the feature map.

Finally, a shuffle operation is performed on the features after channel information capture and spatial information acquisition to fuse the features from different branches, resulting in the final feature representation *I*. The activation function in the ShuffleNetV2 module uses the ReLU function, and the Sigmoid function is used in the SA module. In summary, the SA mechanism achieves effective utilization of channel and spatial information, thereby improving the performance of the network. The head module predicts the object’s feature map, outputting the location of bounding box for the person and behavioral state category of objects.

B. Key-Point Detection Approach

Existing human pose estimation networks can be categorized into two types: one type obtains low-resolution features though high-to-low resolution convolutions and then recovers high-resolution images by progressively up-sampling from encoded low-resolution features. The other type continuously retains high resolution, integrating the multi-scale fusion concept while concurrently interacting with information at different resolutions. Clearly, high resolution maximally preserves the spatial information of image features and provides richer semantic expression. Therefore, for the sensitive issue of detecting key points with inherent variations and complex connection types, HRNet is chosen as the baseline model for key-point detection.

**Figure 6:**
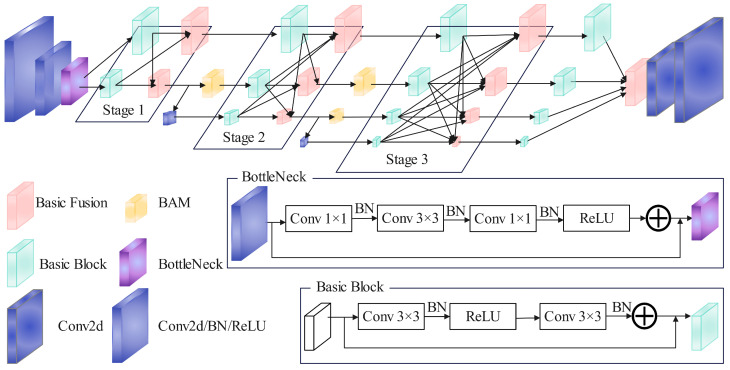
BAM-HRNet network architecture. The structure takes into account both spatial and channel information. On the basis of maintaining the largest-scale feature map, it adds a BAM attention mechanism at other scales to enhance feature extraction capability.

Figure 6 shows the improved HRNet network, which will be referred to as BAM-HRNet in the subsequent description. HRNet achieves strong semantic information and precise positioning by performing information interaction through parallel branches of different resolutions. However, retaining high-resolution features to the maximum extent can cause the network to focus excessively on spatial information while neglecting channel information. For fall detection tasks with high precision requirements, a single feature enhancement structure can reduce the model’s generalization ability. Therefore, after fusing different resolution features, a Bottleneck Attention Module (BAM) is introduced to improve the structure. This module considers both spatial and channel information, enhancing the model’s ability to capture pairwise relationships between pixels and reducing the model’s over-reliance on single-spatial-pixel information.

To reduce the overall size of the network, two 3 × 3 convolutional layers with a stride of 2 are used to decrease the image size to one-quarter of the original. Then, BottleNeck blocks with 1 × 1 convolutions, and multiple activation functions are employed for feature extraction. The module allows more layers to be used with lower computational resources, enabling the Basic Block to apply more non-linear transformations for feature learning. Subsequently, the extracted features are processed through up-sampling and down-sampling operations to obtain features at corresponding resolutions, with Basic Fusion completing the information integration from different receptive fields. Afterward, under the premise of maintaining high resolution, multi-resolution parallel transmission is achieved to facilitate seamless feature information interaction across different scales. At each stage of the BAM-HRNet network, the size description of the characteristic diagram is shown in Table 2.

To mitigate the model’s over-reliance on spatial information, the BAM is integrated during the three-stage transmission, considering both channel and spatial information. This integration enhances the model’s capability to capture pairwise pixel relationships and enables effective learning of more critical information. To preserve the advantages of high-resolution feature extraction, the highest-resolution transmission path in the network remains unaltered, while only features from other scales (excluding the largest scale) undergo channel information exchange. After completing the three stages, features of different scales are fused again via up-sampling to effectively capture key image information. Note that the approach of fusing multi-scale features through up-sampling and down-sampling operations will be elaborated with Algorithm 1.
**Algorithm 1** Multi-scale Feature Fusion Algorithm  1:**Input:** Number of input branches input_branches, Number of output branches output_branches, Number of channels in the first branch *c*  2:**Output:** Fused feature map  3:Initialize list branches  4:**for** i=0 **to** input_branches−1 **do**  5:    Calculate number of channels $w\leftarrow c\times (2^{i})$  6:    Create a sequence containing 4 Basic Blocks and add it to branches  7:**end for**  8:Initialize list fuse_layers as an empty list  9:**for** i=0 **to** output_branches−1 **do**10:    Initialize fuse_layers[i] as an empty list11:    **for** j=0 **to** input_branches−1  **do**12:        **if** i=j **then**13:           Add an Identity operation to fuse_layers[i]14:        **else if** i<j **then**15:           Add 1×1 convolution, with the number of channels changing from $c\times (2^{j})$ to $c\times (2^{i})$ + BN + Up to fuse_layers[i]16:           Scaling factor is $2^{\left(j-i\right)}$17:        **else if** i>j **then**18:           Add 3×3 convolution, with the number of channels remaining the same + BN to fuse_layers[i]19:        **end if**20:    **end for**21:**end for**22:Apply ReLU activation function23:Forward pass (forward):24:**for** each input branch **do**25:    Apply Basic Block to the branch26:**end for**27:Fuse features from each branch28:Process fused features with ReLU activation29:**return** Fused feature map

**Figure 7:**
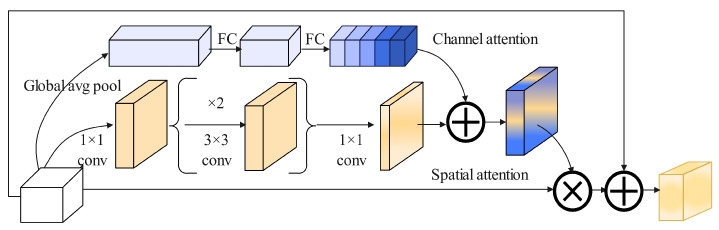
BAM network architecture. The first branch is channel attention, and the second branch is spatial attention. Deep blue channels indicate that channels contain important information, light blue channels are non-important, and deep yellow indicates important spatial information. Then, the enhanced features and the original features are fused.

For BAM [45], Figure 7 illustrates the BAM network structure. it is primarily divided into two branches: channel and spatial attention. In the channel attention branch, the feature map *I* undergoes global average pooling to produce a channel vector $I_{c}\in R^{C\times 1\times 1}$. This vector performs encoding of global information for each channel. To estimate the data of cross-channel from the vector of channels $I_{c}$, MLP with one hidden layer is used.

To reduce parameters, the size of hidden activation is set to $R^{C/r\times 1\times 1}$, where *r* is the reduction ratio. After the MLP, a Batch Normalization (BN) layer [46] is added to adjust the output scale of the spatial branch. The channel attention calculation is Equation (1).

(1) $$I_{c}=BN\left(W_{1}\left(W_{0}AvgPool\left(I\right)+b_{0}\right)+b_{1}\right)$$

The spatial attention branch produces the spatial attention map $I_{s}\in R^{H\times W}$ to emphasize or suppress features at different spatial locations, which helps determine which spatial positions require more focus. Dilated convolutions are used to expand the receptive field. The literature [47] has demonstrated that dilated convolutions are more effective than standard convolutions in constructing spatial feature maps. Specifically, when features $I_{0}\in R^{C\times H\times W}$ are mapped to $R^{C\times H\times W}$, a 1 × 1 convolution is used to aggregate and compress the feature maps along the channel dimension. For convenience, the channel branch uses the same reduction ratio. Subsequently, two 3 × 3 dilated convolutions are applied to leverage contextual information. Finally, a 1 × 1 convolution is used to reduce the features back to the spatial attention map $R^{1\times H\times W}$ to adjust the scale. Batch Normalization layer is applied at the end of the spatial branch. The spatial attention is computed as Equation (2).

(2) $$I_{s}=BN\left(C_{3}^{1\times 1}\left(C_{2}^{3\times 3}\left(C_{1}^{3\times 3}\left(C_{0}^{1\times 1}\left(I\right)\right)\right)\right)\right)$$

where *C* denotes the convolution operation, and BN represents the batch normalization operation. The superscript indicates the size of convolution filters. Channel reduction is performed using two 1 × 1 convolutions, while the intermediate 3 × 3 dilated convolution is used to aggregate contextual information with a larger receptive field.

After obtaining the channel attention $I_{c}$ and spatial attention $I_{s}$ from the two attention branches, we combine them to produce the final 3D attention map $I_{3D}$, Equation (3).

(3) $$I=\sigma \left(I_{c}+I_{s}\right)$$

Due to the different shapes of the two attention maps, they are first expanded to the same shape $R^{C\times H\times W}$ before merging [48,49]. Element-wise addition is chosen to achieve efficient gradient flow. After merging, a Sigmoid function is applied to obtain the final 3D attention map within the range from 0 to 1. Then the 3D attention map is element-wise multiplied with the input feature map *I* and added to the original input frame to obtain the refined feature map $I^{\prime }$, Equation (4).

(4) $$I^{\prime }=I_{0}+I_{0}⊗I$$

#### 3.2.3. Improving Fall Detection Methods

According to key points $\left(x_{i},y_{i}\right)$ and the bounding box $\left(x_{\min},y_{\min},x_{\max},y_{\max}\right)$ of humans to detect falls, $x_{\min}$ and $y_{\min}$ represent the horizontal and vertical coordinates of the top-left corner of the bounding box. $x_{\max}$ and $y_{\max}$ represent the horizontal and vertical coordinates of the bottom-right corner of the bounding box. When a person falls, the aspect ratio of the corresponding bounding box will also change. Therefore, the aspect ratio of the human bounding box can be used as a criterion for detecting fall events. The aspect ratio can be defined by Equation (5).

(5) $$A=\frac{y_{\max}-y_{\min}}{x_{\max}-x_{\min}}$$

When a fall occurs, the center of mass of the human body also continuously descends. The descent rate of the body’s center of mass is defined by Equation (6).

(6) $$V=\frac{\nabla y}{\nabla t}=\frac{y_{initial}-y_{end}}{\nabla t}$$

where $y_{initial}$ is the vertical coordinate of the centroid’s initial position and $y_{end}$ is the vertical coordinate of the centroid’s new position after a period of time ∇t. By using the neck $\left(x_{0},y_{0}\right)$, the centroid $\left(x_{18},y_{18}\right)$, and the projection point of the upright human torso on the ground as the main key points for judgment, the state threshold $\left(x_{19},y_{19}\right)$ that can distinguish between falls and other daily activities is determined by analyzing the coordinate change information of these key points.

The key points numbered 0 and 18 form a planar vector $Z_{1}=\left(x_{18}-x_{0},y_{18}-y_{0}\right)$, and the key points numbered 18 and 19 form another planar vector $Z_{2}=\left(x_{18}-x_{19},y_{18}-y_{19}\right)$. The direction vector $x^{\prime }=\left(1,\;0\right)$ of the *x* axis represents the true direction of the ground. The angle between $x^{\prime }=\left(1,\;0\right)$ and $Z_{1}$, $Z_{2}$ is $\theta_{1}$, $\theta_{2}$. Therefore, the rate of change of the angle Ω between the human torso and the ground is defined by Equation (7).

(7) $$\Omega =\frac{∆\theta_{i}}{∆t}=\frac{\arccos\left(\left[x^{\prime },Z_{i}\right]/\left(∥x^{\prime }∥\cdot ∥Z_{i}∥\right)\right)}{∆t},i=1,2$$

During falling, the values of *A*, *V*, and Ω continuously change [43]. By finding the thresholds that distinguish between different states, it is possible to determine whether a fall has occurred. The classification will be divided into three states: walking, lying, and falling. The detection system is configured to issue an alert exclusively when a fall is detected, indicating an accidental fall event. The fall judgment algorithm is detailed in Algorithm 2.
**Algorithm 2** The fall judgment algorithm  1:**Input:** Video frame sequence, $V_{threshold}=350$, $\Omega_{threshold}=75^{\circ }$  2:**Output:** The status of judgment  3:fall_status←false, judgment_count←0  4:**for** i=1 **to** total_frames **do**  5:    Calculate the aspect ratio *A* of the frame[*i*]  6:    **if** imod15=0 **then**  7:        Calculate centroid velocity *V* and trunk tilt angle Ω of the frame[*i*]  8:        judgment_count←judgment_count+1, previous_A_values.append(*A*)  9:        **if** 1.5<A<2.5 **and** judgment_count≥3 **then**10:           **continue**11:        **end if**12:        **if** A<1 **then**13:           **if** $V>V_{threshold\;}$**and**$\;\Omega >\Omega_{threshold}$ **then**14:               fall_status←true15:               **break**16:           **end if**17:        **end if**18:    **end if**19:    **if** judgment_count=3 **then**20:        **break**21:    **end if**22:**end for**23:**if** fall_status **then**24:    **return** Fall25:**end if**

## 4. Results

### 4.1. Evaluation Metrics

In object detection tasks, evaluating the performance of the model is crucial. To gain a comprehensive understanding of the model’s effectiveness, we analyze and compare the model from different perspectives based on the confusion matrix, as shown in Table 3, including accuracy, precision, recall, and F1-score.

Precision is the proportion of actual positives among the instances predicted as positive by the model, recall is the proportion of actual positives that are correctly predicted by the model, and F1-score is the harmonic mean of precision and recall. The specific formulas are as Equations (8)–(11).

(8) $$precision=\frac{TP}{TP+FP}$$

(9) $$overall\_accuracy=\frac{TP+TN}{TP+TN+FP+FN}$$

(10) $$recall=\frac{TP}{TP+FN}$$

(11) $$F1\_score=2\times \frac{precision\times recall}{precision+recall}=\frac{2TP}{2TP+FN+FP}$$

### 4.2. Model Comparison

To better highlight the advantages of the lightweight network model ShuffleNetV2 and the hybrid attention network model SA, performance comparison experiments were conducted on the ImageNet-1k datasets for common lightweight networks and attention mechanism network models, respectively.

**Figure 8:**
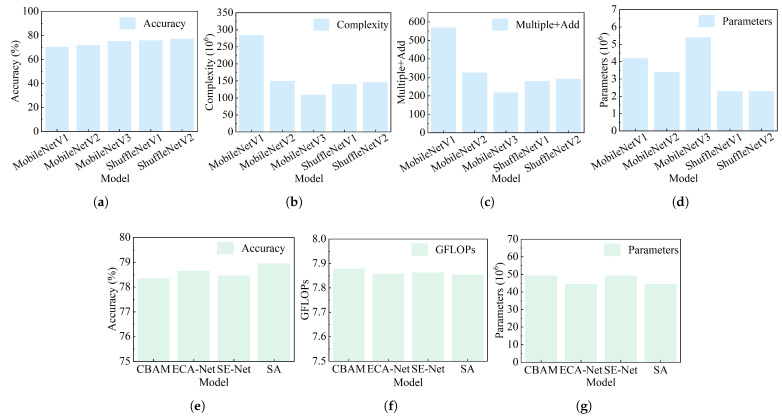
The performance comparison of lightweight network models and attention mechanism network models. The comparison results in the first row correspond to lightweight networks, while those in the second row correspond to attention mechanism networks. (**a**) The comparison results of accuracy. (**b**) The comparison results of complexity. (**c**) The comparison results of multiple + add. (**d**) The comparison results of parameters. (**e**) The comparison results of accuracy. (**f**) The comparison results of GFLOPs. (**g**) The comparison results of parameters.

Figure 8a–d shows the performance comparison results of lightweight network models. ShuffleNetV2 achieves the highest detection accuracy, reaching 77.2%, which is 6.6%, 5.2%, and 2.0% higher than that of MobileNetV1, MobileNetV2, and MobileNetV3, respectively. Although ShuffleNetV2 does not have lower computational complexity and fewer multiply–accumulate operations than MobileNetV3, the number of parameters in the ShuffleNetV2 network is the smallest, with only half of that of the MobileNetV3 model. Additionally, EfficientNet balances accuracy and efficiency through a compound scaling strategy, achieving 82.4% accuracy on the ImageNet datasets with only 5.3 million parameters. However, its training relies on large-scale datasets and powerful computing resources, making it prone to overfitting on small datasets. Additionally, the complex model architecture increases the difficulty of fine-tuning. ResNet addresses the degradation problem of deep networks through residual connections, achieving a detection accuracy of 82.7% on the ImageNet datasets. Although the network has strong generalization ability in transfer learning, its parameter count is extremely large. For example, the ResNet-50 network has 25.6 million parameters, more than 10 times that of ShuffleNetV2, so the deployment of ResNet networks on edge devices is very difficult. Therefore, the reason for choosing ShuffleNetV2 is that it has low computational resource requirements and can balance channel dimensions through 1 × 1 convolutions while effectively utilizing inter-channel connections via channel shuffling. This approach reduces the number of parameters and enhances the network’s feature extraction capability simultaneously.

Figure 8e–g presents the performance comparison results of the attention mechanism network models. The experimental results show that the number of parameters of SA is 4.78 M fewer than that of the CBAM algorithm, while the detection accuracy is increased by 0.606%. The GFLOPs of the SA is 0.025 more than a certain baseline value, reaching 7.854. Compared with the ECA-Net attention mechanism, the number of parameters is the same. However, the detection result of ECA-Net on the ImageNet-1k datasets is 78.650, and the detection accuracy of the SA is 0.31% higher than that of ECA-Net.

### 4.3. Ablation Experiment

Enhance the data and set parameters before training. The input image resolution is 640 × 640 × 3, the initial learning rate is 0.01, the batch size is 16, and the learning rate momentum is 0.937.

**Figure 9:**
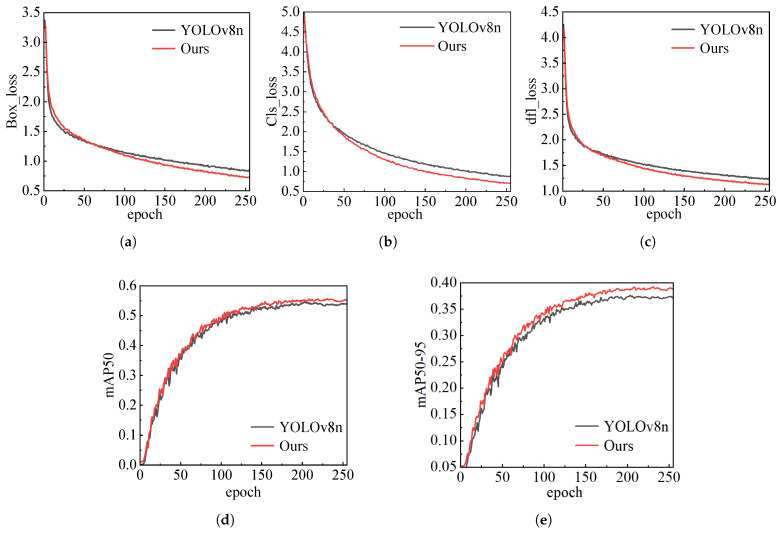
The comparison result of the loss and accuracy of the improved YOLOv8 network in the training process. The graph in the first row is the loss change graph, and the graph in the second row is the result graph of mAP. (**a**) The loss of the boundary box. (**b**) The loss of classification. (**c**) The total loss. (**d**) The result of mAP50. (**e**) The result of mAP50–95.

In order to verify the effectiveness of the improved algorithm, this study conducted modular ablation experiments on the network. Figure 9 shows the comparison of the loss and map accuracy detection results between the improved YOLOv8 and the baseline model during training. Figure 9a–c show the bounding-box loss, target classification loss, and total loss of human positioning. Figure 9d,e show the results of mAP50 and mAP50–95, respectively. During training, the improved network has lower loss values for bounding-box, classification, and total loss compared to the YOLOv8, while the mAP value is higher than the baseline, which can effectively achieve human localization.

Table 4 shows the results of the ablation study on the improved YOLOv8 model. On the COCO dataset, the precision reached 64.1%, representing a 0.8% improvement. The mAP@0.5, 0.75, and 0.5:0.95 values are 53.4%, 49.0%, and 38.5%, respectively, showing 0.9%, 0.4%, and 1.3% improvements over the baseline model. On the Pascal VOC dataset, precision achieved 61.7%, an increase of 1.0%. These results demonstrate that the lightweight depth-wise separable convolutions, channel information shuffling operations in ShuffleNetV2, and the mixed attention mechanism not only effectively reduce the computational load of the backbone network but also significantly enhance detection accuracy.

Table 5 shows the results of the ablation study on the improved HRNet key-point extraction network. On the COCO datasets, the precision of the baseline at IOU thresholds of 0.50, 0.75, and 0.50:0.95 was 92.32%, 79.92%, and 70.14%, respectively, while the recall rates at the same IOU thresholds were 93.09%, 82.62%, and 76.21%. The most significant improvement was seen at the IOU threshold of 0.50:0.95, with an increase of 3.35%. On the OCHuman datasets, the mAP@0.50 exhibited the most substantial improvement, reaching 86.34%, which represents an increase of 1.59%. This indicates that the proposed algorithm has made significant improvements in object detection tasks and has enhanced the model’s ability to localize human bodies in the elderly fall detection task.

### 4.4. Comparable Experiments

The comparative experiments are divided into two parts: one is a comparison of object detection algorithms, and the other is a comparison of key-point detection algorithms.

To further validate the effectiveness of the improved YOLOv8 network, the experiment compared the backbone, parameters, GFLOPs, speed, and mAP between YOLO series models [31,42,43,44,45,46]. Table 6 shows the results of performance comparison of YOLO series models. The models involved in the comparison include YOLOv5 [51], YOLOv7 [41], YOLOv8 [52], YOLOv9 [53], YOLOv10 [54], and YOLOv11 [55]. The experimental results show that compared to YOLOv5s, the improved network has increased the parameter count by 2.1M and the GFLOPs by 8.2, with a slight increase of 1.5ms in latency. However, in detection tasks, the proposed network achieves a 7% improvement in mAP@0.50 and a 7.6% improvement in mAP@0.50:0.95 over YOLOv5s. The YOLOv7 series exhibits overall higher detection accuracy than the proposed network due to its more complex model with parameters 6.8 times and 5.4 times more than the proposed model. Compared to YOLOv8s and YOLOv9T, the proposed network improves mAP@0.50 by 1.5% and 0.3%, respectively. Clearly, YOLOv9C performs slightly better than the proposed model in terms of precision, which is attributed to YOLOv9’s backbone network. But, the memory requirement of YOLOv9 during training is still higher than that during inference due to the need to save extra intermediate activation. Compared with YOLOv10 and YOLOv11, the proposed algorithm has fewer parameters, but the detection accuracy is equivalent. The backbone network utilizes a general efficient layer aggregation network in the gradient path, employing traditional convolution operators for feature extraction and handling various changes in multiple targets through programmable gradient information. However, the use of traditional convolution operators inevitably increases the complexity of the model.

Table 7 shows the comparison results of the detection performance between the proposed algorithm and other existing algorithms on the COCO datasets. Compared with the ViDT algorithm, they have the same number of parameters, and the detection accuracy of the algorithm proposed in this paper is slightly better. However, the proposed algorithm performs poorly in terms of real-time performance.

**Figure 10:**
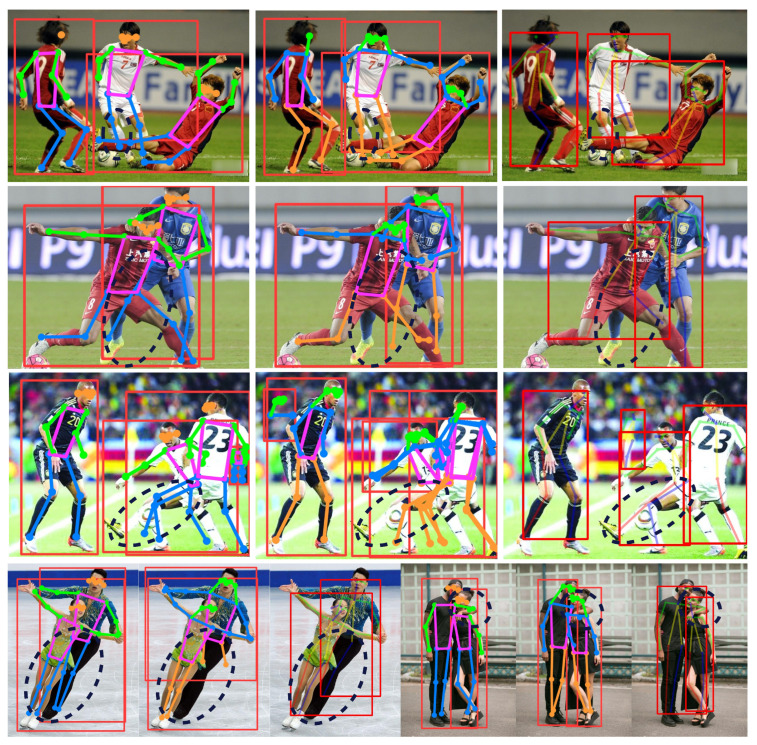
The visual results of the proposed method compared. The left shows the visualization detection results of the proposed algorithm in this paper, the middle shows the results of YOLOv8, and the right shows the results of Lightweight OpenPose.

Figure 10 shows the visual results of the proposed method compared to YOLOv8s-pose and Lightweight OpenPose on OCHuman with occluded images. The left side of the image displays the results predicted by the proposed method, the middle shows the results from YOLOv8s-pose, and the right side presents the predictions from Lightweight OpenPose [19].

Clearly, the proposed method provides more accurate predictions for some occluded key points, with differences highlighted by black dashed ellipses in the detection results. In the first row, YOLOv8s-pose fails to accurately locate the key points of a person in white clothing bending their knee, while Lightweight OpenPose performs better. However, the proposed method shows comparable accuracy to Lightweight OpenPose. In the second row, the proposed method correctly identifies the occluded leg of an athlete in blue, whereas the other two algorithms fail. In the third row, after the leg of a white-clad athlete is partially obscured by a soccer ball, both the proposed method and YOLOv8s-pose exhibit detection shifts, while Lightweight OpenPose accurately locates the leg. In the fourth row, the first set of visualized data shows that only the proposed method detects all key points of a male dancer accurately, while the other two methods fail to recognize them. In the second set of visualized data in the fourth row, all three algorithms accurately identify human body key points. In summary, although the proposed algorithm may have slight deviations in detecting occluded key points—especially when the subject wears light-colored clothing and is obscured by similarly colored objects—it outperforms other algorithms overall in human pose estimation under occluded scenarios. This method enhances the detection accuracy of occluded key points, which is more conducive to accurately identifying fall conditions.

Table 8 shows the accuracy comparison results of key-point extraction algorithms. The proposed network architecture shows a notable improvement on the COCO key-point datasets. The precision of AP@0.50 reaches 92.48%, ranking second among the compared algorithms, only 0.05% lower than the top-ranking algorithm. For AP@0.75, the proposed network achieves 81.36%, ranking first. The mAR@0.5 and mAR@0.75 are 93.45% and 83.11%, respectively. In terms of the precision represented by AP@0.50 and AP@0.75 and the recall represented by mAR@0.5 and mAR@0.75, the proposed network improves the precision of the base model HRNet by 0.16% and 0.36%, respectively. This indicates that by simultaneously considering spatial and channel features in the original network structure, the network enhances its ability to learn features, further improving the accuracy of key-point extraction and laying a foundation for subsequent fall detection.

**Figure 11:**
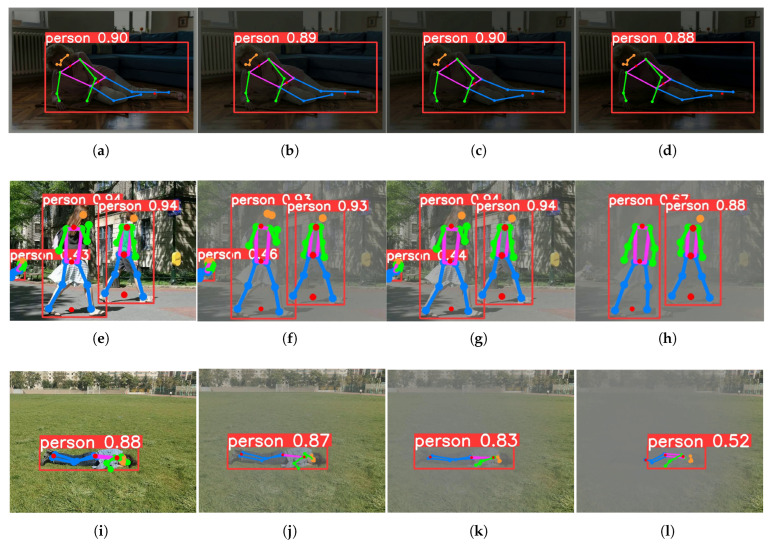
Visualization results of key-point detection under different light intensities. The first line shows the detection results under different lighting conditions, while the second and third lines show the key-point detection results under different levels of haze. The difference is that the second line adds fog to the image by setting the peak signal-to-noise ratio (PSNR), while the third line adds fog to the image by setting different fog concentrations. The parameter settings are as follows: (**a**) 0.05, (**b**) 0.04, (**c**) 0.03, (**d**) 0.01, (**e**) original, (**f**) 5, (**g**) 15, (**h**) 25, (**i**) original, (**j**) 0.01, (**k**) 0.03, (**l**) 0.06.

Figure 11 shows the visualization results of key-point detection under different light intensities. From the detection results, it can be seen that the proposed algorithm can effectively extract key points of the human body in both dimly lit indoor and foggy outdoor environments.

### 4.5. Fall Detection

#### Fall Threshold Analysis

In order to obtain accurate fall discrimination thresholds, 65 frames of video sequences were captured for three behavioral states: falling, lying down, and walking. For each video sequence, the human body aspect ratio, centroid variation, and torso tilt angle were calculated.

**Figure 12:**
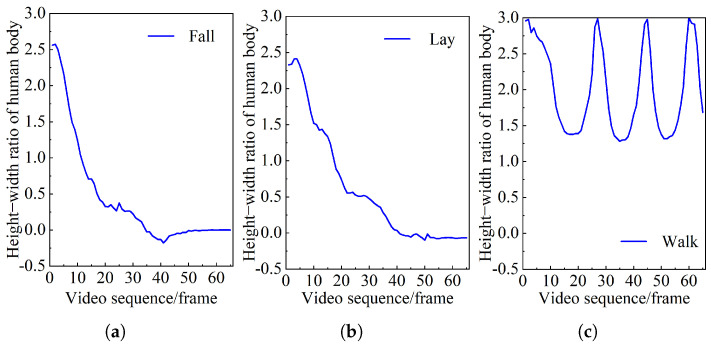
Height/width ratio of human body. (**a**) Falling, (**b**) lying, and (**c**) walking.

Figure 12 shows a comparison of the changes in body features for different behavioral states. By observing the curve, when 1.5≤A<3, the height-to-width ratio of normal walking behavior exhibits a regular periodic fluctuation every 15 frames. However, when A≤1, the lying behavior in normal human activities is very similar to the height/width ratio after falling, which cannot be judged by setting the ratio threshold. However, by observing the curve between 0 and 40 frames, it is found that the falling speed of the numerical change curve of the height/width ratio of the human body in the lying behavior is slower than that in the falling process. This is because the centroid descent speed during uncontrolled accidental falls is faster than that in daily behaviors.

**Figure 13:**
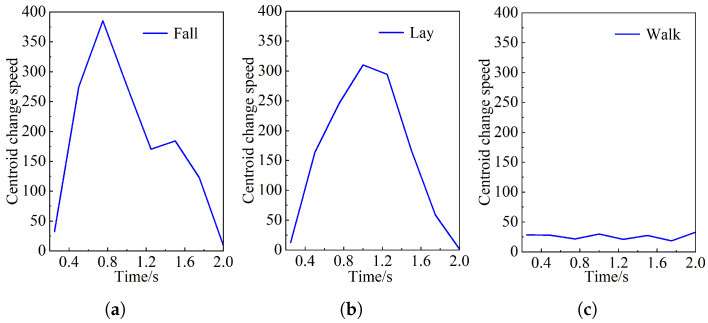
Centroid change speed of human body. (**a**) Falling, (**b**) lying, and (**c**) walking.

Figure 13 shows the comparison of the falling speed of the center of mass in five different behavior states after each 0.25 s. Observe the peak value of the decline speed of the center of mass in the five behaviors. When falling, the falling speed of the center of mass exceeds 350 pixels per second. Therefore, the falling discrimination threshold of the falling speed of the center of mass is set to 350.

When the human body completes different behavior activities, the torso of the human body will also tilt. According to the statistics of the changing speed of the inclination of the human trunk vector $Z_{1}$ and $Z_{2}$ of 50 subjects with different behaviors, the angle between the trunk and the horizontal direction will change by more than 75°. Therefore, when the trunk inclination angle changes to 75°, it is judged that the human body being tested falls.

**Figure 14:**
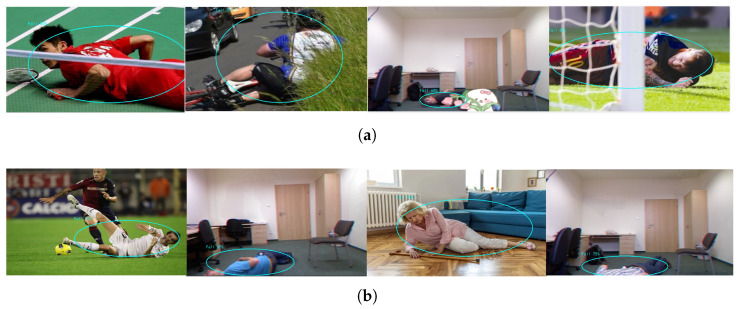
The results of fall detection. The circles represent the visual representation of fall events detected by the system. (**a**) Falling detection results with occlusion, (**b**) the results of indoor fall detection.

Figure 14 shows the results of detection on the public datasets (UR Fall Detection [37], Fall Detection datasets [36]), indicating that the proposed algorithm can effectively detect falls. The algorithm achieves an accuracy of 95.0% on the human fall datasets.

## 5. Discussion and Conclusions

This paper proposes a top-down fall detection algorithm that fuses the improved YOLOv8 and BAM-HRNet to extract human skeletal key points, effectively addressing the issues of insufficient feature extraction, single-fall judgment criterion, and poor real-time performance in occluded scenarios. First, the algorithm enhances the YOLOv8 network by integrating ShuffleNetV2 and the SA attention mechanism to improve human body positioning accuracy. It then employs BAM-HRNet to effectively extract human key points. By analyzing the positional change information of dynamic skeletal key points and identifying abnormal fall patterns, the algorithm improves fall detection accuracy. Experimental results show that the object detection module achieves accuracies of 64.1% and 61.7% on the COCO and Pascal VOC datasets, respectively, while the key-point detection module attains 73.49% and 70.11% on the COCO and OCHuman datasets. Ultimately, the algorithm demonstrates an accuracy of over 95% on human fall datasets. Compared with other methods, the proposed approach is more precise and effective in distinguishing between normal activities and accidental falls. However, the algorithm still has room for optimization—for example, improving its real-time performance is a research direction for future work.

## Figures and Tables

**Table 1:** Comparison results of group training on the COCO datasets. Key points are grouped into ‘The whole body’, ‘The upper/lower half of the body,’ and ‘head/arm/body/leg’ categories. The results show that the ‘head/arm/body/leg’ categories yield the best performance.

Grouping Type	Precision	Recall	F1-Score
The whole body	73.06	75.03	74.04
The upper/lower half of the body	73.12	75.47	74.28
Head/arm/body/leg	73.49	76.51	74.97

**Table 2:** In each stage of the BAM-HRNet network, the size description of the feature map.

Branch	Transition1	Stage1	Transition2	Stage2	Transition3	Stage3	Number of Channels
1	64×48	64×48	64×48	64×48	64×48	64×48	32
2	32×24	32×24	32×24	32×24	32×24	32×24	64
3	-	-	16×12	16×12	16×12	16×12	128
4	-	-	-	-	8×6	8×6	256

**Table 3:** Confusion matrix.

	Predicted Label	
	True	False
Ground-Truth True	TP (True Positive)	FN (False Negative)
Ground-Truth False	FP (False Positive)	TN (True Negative)

**Table 4:** The results of the ablation study on the improved YOLOv8 model. The experimental datasets use COCO and Pascal VOC.

Datasets	YOLOv8n	ShuffleNetV2	SA	Precise	Recall	mAP		
						0.50	0.75	0.50:0.95
COCO [33]	✓			63.3	48.0	52.5	48.6	37.2
	✓	✓		62.7	47.4	52.2	47.3	36.9
	✓		✓	64.2	48.6	53.0	48.9	37.2
	✓	✓	✓	64.1	48.8	53.4	49.0	38.5
Pascal VOC [34]	✓			60.7	45.7	51.5	49.7	30.1
	✓	✓		60.4	46.6	50.0	49.6	30.6
	✓		✓	61.8	46.2	54.6	50.1	33.7
	✓	✓	✓	61.7	46.3	63.7	50.4	30.7

**Table 5:** The results of the ablation study on the improved BAM-HRNet model. The experimental datasets use COCO and OCHuman.

Datasets	HRNet	BAM	Precision			Recall		
			0.50	0.75	0.50:0.95	0.50	0.75	0.50:0.95
COCO [33]	✓		92.32	79.92	70.14	93.09	82.62	76.21
	✓	✓	92.48	81.36	73.49	93.45	83.11	76.51
OCHuman [35,50]	✓		84.75	72.36	69.38	84.89	73.42	70.33
	✓	✓	86.34	73.59	70.11	87.61	75.59	70.12

**Table 6:** Performance comparison results of object detection models in YOLO series.

Model	Backbone	Parameters	GFLOPs	Speed/ms	mAP	
					0.50	0.50:0.95
YOLOv5n	CSPDarknet-53	1.8 M	4.5	12.7	45.1	27.7
YOLOv5s	CSPDarknet-53	7.2 M	16.5	13.0	55.2	37.1
YOLOv5m	CSPDarknet-53	21.2 M	49.0	16.8	63.5.1	45.0
YOLOv5l	CSPDarknet-53	46.5 M	109.1	25.6	66.8	48.8
YOLOv5x	CSPDarknet-53	86.7 M	205.7	27.1	68.3	50.4
YOLOv7	ELAN	36.9 M	104.5	21.0	69.1	49.6
YOLOv7x	ELAN	71.3 M	189.0	39.6	70.2	51.4
YOLOv8n	Darknet-53	3.2 M	8.7	14.8	52.5	37.3
YOLOv8s	Darknet-53	11.2 M	28.6	13.9	61.6	44.5
YOLOv8m	Darknet-53	25.9 M	78.9	20.5	67.0	50.1
YOLOv8l	Darknet-53	43.7 M	165.2	30.7	69.7	52.9
YOLOv8x	Darknet-53	68.2 M	257.8	36.2	70.9	54.0
YOLOv9T	GELAN	2.0 M	7.7	12.7	53.1	38.1
YOLOv9S	GELAN	7.1 M	26.4	17.6	63.4	46.8
YOLOv9 M	GELAN	20.0 M	76.3	31.9	67.9	51.3
YOLOv9C	GELAN	25.3 M	102.1	42.6	70.1	53.0
YOLOv9E	GELAN	58.1 M	192.5	51.4	72.7	55.6
YOLOv10N	SC Down	2.7 M	6.7	12.0	53.2	38.1
YOLOv10S	SC Down	8.1 M	21.6	13.4	62.5	45.8
YOLOv10 M	SC Down	16.5 M	59.1	19.7	67.5	50.6
YOLOv10B	SC Down	20.5 M	92.0	21.6	69.0	51.9
YOLOv10L	SC Down	25.8 M	120.3	25.8	69.5	52.6
YOLOv10X	SC Down	31.7 M	160.4	39.4	70.8	53.9
YOLOv11N	C3K2	2.6 M	6.5	11.6	54.9	39.3
YOLOv11S	C3K2	9.4 M	21.5	13.5	63.4	46.8
YOLOv11 M	C3K2	20.1 M	68.0	19.3	67.9	51.5
YOLOv11L	C3K2	25.3 M	86.9	20.6	69.5	53.4
YOLOv11X	C3K2	56.9 M	194.9	45.9	71.5	53.9
Ours	ShuffleNetV2	7.0 M	17.8	12.7	53.4	38.5

**Table 7:** Performance comparison results of object detection models.

Model	Backbone	Parameters	FPS	mAP	
				0.50	0.50:0.95
DETR [56]	Deit-base	104 M	10.1	59.2	37.1
ViDT [57]	Swin-nano	7 M	21.3	48.6	28.7
DETR-DC5	ResNet-50	41 M	13.5	55.7	35.3
Ours	ShuffleNetV2	7 M	20.6	53.4	38.5

**Table 8:** The comparison results of key-point extraction algorithms.

Model	AP					AR				
	0.50	0.75	0.50:0.95			0.50	0.75	0.50:0.95		
			All	Medium	Large			All	Medium	Large
HRNet [32]	92.32	79.92	72.72	70.14	76.72	93.09	82.62	76.21	72.85	81.32
CABM-HRNet [58]	92.49	80.53	72.97	70.11	77.43	93.20	82.43	75.99	72.76	80.90
Deep-HRNet [59]	92.47	80.50	73.57	70.72	78.46	93.37	82.95	76.66	73.27	81.83
ECA-HRNet [60]	91.48	79.07	71.14	68.22	75.42	92.51	81.03	74.30	71.10	79.15
Coord-HRNet [61]	92.53	80.43	73.19	70.46	77.31	93.40	82.84	76.15	72.97	80.98
HRFormer-B [62]	90.86	82.89	75.63	71.76	82.65	91.73	83.01	75.46	71.56	80.87
TransPose-H/A6 [63]	90.19	82.13	75.84	71.92	82.81	91.21	83.37	80.79	72.35	82.03
TokenPose-L/D24 [64]	90.30	82.52	75.83	72.34	82.75	92.41	81.42	74.59	71.36	80.93
DEKR [56]	88.33	78.61	72.34	68.62	78.65	87.58	79.17	77.71	69.14	78.85
Light OpenPose [19]	81.22	63.34	60.67	66.14	67.65	80.88	62.35	59.79	62.35	64.05
OpenPose [24]	84.91	67.54	61.82	62.33	69.24	85.18	67.38	60.45	63.36	65.96
AlphaPose [58]	81.33	66.04	61.13	61.45	56.57	82.13	66.51	60.45	60.75	64.37
Ours	92.48	81.36	73.49	70.60	78.08	93.45	83.11	76.51	73.14	81.58

## Data Availability

The data presented in this study are available on request from the corresponding author.

## References

[1] World Health Organization. https://www.who.int/news-room/fact-sheets/detail/falls/.

[2] Shahzad A., Kiseon K. (2018). FallDroid: An automated smart-phone-based fall detection system using multiple kernel learning. IEEE Trans. Ind. Inform..

[3] Bharathi V., Dhakne A., Preethi P. (2023). AI based elderly fall prediction system using wearable sensors: A smart home-care technology with IOT. Meas. Sens..

[4] Ning Y., Hu S., Nie X., Liang S., Li H., Zhao G. Real-time action recognition and fall detection based on smartphone. Proceedings of the 2018 40th Annual International Conference of the IEEE Engineering in Medicine and Biology Society (EMBC).

[5] Maray N., Ngu A.H., Ni J., Debnath M., Wang L. (2023). Transfer learning on small datasets for improved fall detection. Sensors.

[6] Ngu M.N., Metsis A.H., Coyne V., Srinivas P., Salad T., Mahmud U., Chee K.H. (2022). Personalized watch-based fall detection using a collaborative edge-cloud framework. Int. J. Neural Syst..

[7] Ning Y., Hu S., Nie X., Liang S., Li H., Zhao G. (2018). SmartFall: A smartwatch-based fall detection system using deep learning. Sensors.

[8] Şengül G., Karakaya M., Misra S., Abayomi-Alli O.O., Damaševičius R. (2022). Deep learning based fall detection using smartwatches for healthcare applications. Biomed. Signal Process. Control.

[9] Mohan D., Al-Hamid D.Z., Chong P.H.J. (2024). Artificial intelligence and IOT in elderly fall prevention: A review. IEEE Sens. J..

[10] Wang P., Li Q., Yin P., Wang Z., Ling Y., Gravina R., Li Y. (2023). A convolution neural network approach for fall detection based on adaptive channel selection of UWB radar signals. Neural Comput. Appl..

[11] Islam M.A., Kowal M., Jia S., Derpanis K.G., Bruce N.D.B. (2024). Position, padding and predictions: A deeper look at position information in cnns. Int. J. Comput. Vis..

[12] Zhou X., Qian L.-C., You P.-J., Ding Z.-G., Han Y.-Q. Fall detection using convolutional neural network with multi-sensor fusion. Proceedings of the 2018 IEEE International Conference on Multimedia Expo Workshops (ICMEW).

[13] Graves A., Graves A. (2012). Long short-term memory. Supervised Sequence Labelling with Recurrent Neural Networks.

[14] Liu G.S., Xiong P. (2015). Human fall detection based on three feature points. Technol. Vis..

[15] Nogas J., Khan S.S., Mihailidis A. (2020). Deepfall: Non-invasive fall detection with deep spatio-temporal convolutional autoencoders. J. Healthc. Inform. Res..

[16] Shu Y.X., Lei H. (2023). A vision-based human posture detection approach for smart home applications. Int. J. Adv. Comput. Sci. Appl..

[17] Rougier C., Meunier J., Arnaud A., Rousseau J. Fall detection from human shape and motion history using video surveillance. Proceedings of the 21st International Conference on Advanced Information Networking and Applications Workshops (AINAW).

[18] Yuan B.L., Wang Y.H., Wang X. (2023). A computer vision monitoring for human fall using visible light camera and thermal imager. Int. J. Model. Identif. Control.

[19] Wang X., Liu X.N., Gao H.B., Zeng Z.M., Zhang Y.L. (2024). Workshop worker fall detection algorithm based on visible light thermal infrared visual monitoring. Control Decis..

[20] Han G.T., Wang H., Wang S., Chen E., Wang G. (2024). Joint keypoint data augmentation and structural prior for occlusion human pose estimation. Comput. Eng. Appl..

[21] Targ S., Almeida D., Lyman K. (2016). Resnet in resnet: Generalizing residual architectures. arXiv.

[22] Simonyan K., Zisserman A. (2014). Very deep convolutional networks for large-scale image recognition. arXiv.

[23] Cao Z., Hidalgo G., Simon T., Wei S.E., Sheikh Y. Realtime multi-person 2d pose estimation using part affinity fields. Proceedings of the IEEE Conference on Computer Vision and Pattern Recognition.

[24] Cao Z., Hidalgo G., Simon T., Wei S.E., Sheikh Y. Openpose: Realtime multi-person 2d pose estimation using part affinity fields. Proceedings of the IEEE Transactions on Pattern Analysis and Machine Intelligence.

[25] Bian Z.P., Chau L.P., Thalmann N.M. Fall detection based on skeleton extraction. Proceedings of the 11th ACM SIGGRAPH International Conference on Virtual-Reality Continuum and its Applications in Industry.

[26] Insafutdinov E., Pishchulin L., Andres B., Andriluka M., Schiele B. Deepercut: A deeper, stronger, and faster multi-person pose estimation model. Proceedings of the Computer Vision–ECCV 2016: 14th European Conference.

[27] Angelini F., Fu Z., Long Y., Shao L., Naqvi S.M. (2019). 2D pose-based real-time human action recognition with occlusion-handling. IEEE Trans. Multimed..

[28] Raza A., Yousaf M.H., Velastin S.A. Human fall detection using YOLO: A real-time and AI-on-the-edge perspective. Proceedings of the 2022 12th International Conference on Pattern Recognition Systems (ICPRS).

[29] Bo L.U.O. (2023). Human fall detection for smart home caring using yolo networks. Int. J. Adv. Comput. Sci. Appl..

[30] Wang B.H., Yu J., Wang K., Bao X.-Y., Mao K.-M. (2020). Fall detection based on dual-channel feature integration. IEEE Access.

[31] Wang J., Sun K., Cheng T., Jiangh B., Deng C., Zhao Y., Mu Y., Tan M., Wang X., Liu W. (2020). Deep high-resolution representation learning for visual recognition. IEEE Trans. Pattern Anal. Mach. Intell..

[32] Wang J., Qin Q., Li Z., Ye X., Wang J., Yang X. Deep hierarchical representation and segmentation of high resolution remote sensing images. Proceedings of the 2015 IEEE International Geoscience and Remote Sensing Symposium (IGARSS).

[33] Lin T.Y., Maire M., Belongie S., Hays J., Perona P., Ramanan D., Dollár P., Zitnick C.L. Microsoft coco: Common objects in context. Proceedings of the Computer Vision–ECCV 2014: 13th European Conference.

[34] Everingham M., Gool L.V., Williams C.K.I. The PASCAL Visual Object Classes Challenge 2012 (VOC2012) Results. http://www.pascal-network.org/challenges/VOC/voc2012/workshop/index.html.

[35] Zhang S.-H., Li R., Dong X., Rosin P., Cai Z., Han X., Yang D., Huang H., Hu S.-M. (2018). Pose2seg: Human instance segmentation without detection. arXiv.

[36] Adhikari K., Bouchachia H. Activity recognition for indoor fall detection using convolutional neural network. Proceedings of the 2017 Fifteenth IAPR International Conference on Machine Vision Applications (MVA).

[37] Kwolek B., Kepski M. (2014). Human fall detection on embedded platform using depth maps and wireless accelerometer. Comput. Methods Programs Biomed..

[38] Zhong Z., Zheng L., Kang G., Li S., Yang Y. Random erasing data augmentation. Proceedings of the AAAI Conference on Artificial Intelligence.

[39] Yang K.Y., Qinami K. Towards fairer datasets: Filtering and balancing the distribution of the people subtree in the imagenet hierarchy. Proceedings of the 2020 Conference on Fairness, Accountability, and Transparency.

[40] Ma N.N., Zhang X., Zheng H.T., Sun J. Shufflenet v2: Practical guidelines for efficient cnn architecture design. Proceedings of the European Conference on Computer Vision (ECCV).

[41] Wang C.Y., Bochkovskiy A. YOLOv7: Trainable bag-of-freebies sets new state-of-the-art for real-time object detectors. Proceedings of the IEEE/CVF Conference on Computer Vision and Pattern Recognition.

[42] He K., Zhang X., Ren S., Sun J. (2015). Spatial pyramid pooling in deep convolutional networks for visual recognition. IEEE Trans. Pattern Anal. Mach. Intell..

[43] Wang X.P., Shi H. (2024). Improved yolov8 fall detection algorithm based on fusion of key points. J. Xi’An Univ. Electron. Sci. Technol..

[44] Zhang Q.L., Yang Y.B. Sa-net: Shuffle attention for deep convolutional neural networks. Proceedings of the ICASSP 2021–2021 IEEE International Conference on Acoustics, Speech and Signal Processing (ICASSP).

[45] Park J., Woo S., Lee J.Y., Kweon I.S. (2018). Bam: Bottleneck attention module. arXiv.

[46] Peerthum Y., Mark S. (2023). An empirical analysis of the shift and scale parameters in BatchNorm. Inf. Sci..

[47] Chen X., Fang H., Lin T.Y., Vedantam R., Gupta S., Dollar P., Zitnick C.L. (2015). Microsoft coco captions: Data collection and evaluation server. arXiv.

[48] Rassekh E., Snidaro L. (2025). Survey on data fusion approaches for fall-detection. Inf. Fusion.

[49] Almukadi W.S., Alrowais F., Saeed M.K. (2024). Deep feature fusion with computer vision driven fall detection approach for enhanced assisted living safety. Sci. Rep..

[50] Zhang S.-H., Li R., Dong X., Rosin P., Cai Z., Han X., Yang D., Huang H., Hu S.-M. Pose2seg: Detection free human instance segmentation. Proceedings of the IEEE/CVF Conference on Computer Vision and Pattern Recognition.

[51] Zhu X.H., Lu S., Wang X. TPH-YOLOv5: Improved YOLOv5 based on transformer prediction head for object detection on drone-captured scenarios. Proceedings of the IEEE/CVF International Conference on Computer Vision.

[52] Lakshmi N.S., Ghosh S., Das S.K. Deep Learning-Based Flying Object Detection using Avian-Airborne Database: Military Applications. Proceedings of the 2023 IEEE 20th India Council International Conference (INDICON).

[53] Wang C.Y., Yeh I.H., Liao H.Y.M. Yolov9: Learning what you want to learn using programmable gradient information. Proceedings of the European Conference on Computer Vision.

[54] Wang A., Chen H., Liu L., Chen K., Lin Z., Han J., Ding G. (2025). Yolov10: Real-time end-to-end object detection. Adv. Neural Inf. Process. Syst..

[55] Khanam R., Hussain M. (2024). Yolov11: An overview of the key architectural enhancements. arXiv.

[56] Geng Z.G., Sun K., Xiao B., Zhang Z., Wang J. Bottom-up human pose estimation via disentangled keypoint regression. Proceedings of the IEEE/CVF Conference on Computer Vision and Pattern Recognition.

[57] Wang H.X., Zhou L., Chen Y.Y., Tang M., Wang J. (2024). EFCPose: End-to-end multi-person pose estimationWith fully convolutional heads. IEEE Trans. Circuits Syst. Video Technol..

[58] Woo S., Park J., Lee J.-Y., Kweon I.S. Cbam: Convolutional block attention module. Proceedings of the European Conference on Computer Vision (ECCV).

[59] Zhang H., Dun Y., Pei Y., Lai S., Liu C., Zhang K., Qian X. (2024). HF-HRNet: A simple hardware friendly high-resolution network. IEEE Trans. Circuits Syst. Video Technol..

[60] Wang Q., Wu B., Zhu P., Li P., Zuo W., Hu Q. ECA-Net: Efficient channel attention for deep convolutional neural networks. Proceedings of the IEEE/CVF Conference on Computer Vision and Pattern Recognition.

[61] Hou Q.B., Zhou D.Q., Feng J.S. Coordinate attention for efficient mobile network design. Proceedings of the IEEE/CVF Conference on Computer Vision and Pattern Recognition.

[62] Yuan Y.H., Fu R., Huang L. (2021). Hrformer: High-resolution transformer for dense prediction. arXiv.

[63] Yang S., Quan Z., Nie M., Yang W. Transpose: Keypoint localization via transformer. Proceedings of the IEEE/CVF International Conference on Computer Vision.

[64] Li Y., Zhang S., Wang Z., Yang S., Yang W., Xia S.-T., Zhou E. Tokenpose: Learning keypoint tokens for human pose estimation. Proceedings of the IEEE/CVF International Conference on Computer Vision.

